# An Accelerated Release Study to Evaluate Long-Acting Contraceptive Levonorgestrel-Containing in Situ Forming Depot Systems

**DOI:** 10.3390/pharmaceutics8030028

**Published:** 2016-09-01

**Authors:** Dileep R. Janagam, Lizhu Wang, Suryatheja Ananthula, James R. Johnson, Tao L. Lowe

**Affiliations:** Department of Pharmaceutical Sciences, University of Tennessee Health Sciences Center, Memphis, TN 38163, USA; djanagam@uthsc.edu (D.R.J.); lwang77@uthsc.edu (L.W.); sananthu@uthsc.edu (S.A.); jjohns42@uthsc.edu (J.R.J.)

**Keywords:** in situ forming depot system, in situ implant, injectable, accelerated release, poly(lactide-*co*-glycolide), polylactic acid, solvents, levonorgestrel, correlation

## Abstract

Biodegradable polymer-based injectable in situ forming depot (ISD) systems that solidify in the body to form a solid or semisolid reservoir are becoming increasingly attractive as an injectable dosage form for sustained (months to years) parenteral drug delivery. Evaluation of long-term drug release from the ISD systems during the formulation development is laborious and costly. An accelerated release method that can effectively correlate the months to years of long-term release in a short time such as days or weeks is economically needed. However, no such accelerated ISD system release method has been reported in the literature to date. The objective of the current study was to develop a short-term accelerated in vitro release method for contraceptive levonorgestrel (LNG)-containing ISD systems to screen formulations for more than 3-month contraception after a single subcutaneous injection. The LNG-containing ISD formulations were prepared by using biodegradable poly(lactide-*co*-glycolide) and polylactic acid polymer and solvent mixtures containing *N*-methyl-2-pyrrolidone and benzyl benzoate or triethyl citrate. Drug release studies were performed under real-time (long-term) conditions (PBS, pH 7.4, 37 °C) and four accelerated (short-term) conditions: (A) PBS, pH 7.4, 50 °C; (B) 25% ethanol in PBS, pH 7.4, 50 °C; (C) 25% ethanol in PBS, 2% Tween 20, pH 7.4, 50 °C; and (D) 25% ethanol in PBS, 2% Tween 20, pH 9, 50 °C. The LNG release profile, including the release mechanism under the accelerated condition D within two weeks, correlated (*r*^2^ ≥ 0.98) well with that under real-time conditions at four months.

## 1. Introduction

An injectable in situ forming depot/implant (ISD) system is a solution or suspension containing drug and biodegradable polymers dissolved or suspended in pharmaceutically acceptable water-miscible organic solvents, respectively [1,2,3,4,5,6]. Upon injection into the target tissue, the ISD forms a solid depot of polymeric matrix at the injection site because of the phase separation by solvent exchange, as the water-miscible organic solvent diffuses into the surrounding aqueous medium while the aqueous body fluid penetrates into the organic phase [3,4,7,8]. The drug entrapped in the depot is then slowly released out into the surrounding body fluid due to the degradation of the polymers and diffusion of the drug, goes into the systemic circulation and then reaches target sites. The biodegradable polymers will eventually completely degrade at the injection site over a period of time, and be cleared out from the body. Because of the complete biodegradation, the ISD system does not need any surgical removal at the end of the treatment. The ISD technology has been developed aiming at safe and efficient delivery of drugs in vivo for over periods of time ranging from weeks to months [2,4,6,9], to treat cancer, infections, hormonal disorders, pain, immunomodulation, neurological disorders and metabolic disorders [4,10]. Other advantages of the system include simplicity and low cost of manufacturing, ease of application to the body, and versatility of the technology [6]. Several ISD systems are currently available, such as the products marketed as Eligard^®^ and Atridox^®^ [4].

During the early stage formulation development of ISD systems, it is important and relatively economical to conduct in vitro drug release to prescreen/differentiate the formulations, and identify critical parameters for optimizing the formulations to achieve the desired drug release profiles before more expensive animal validation. However, even within the in vitro evaluation, the real-time release under physiological conditions (PBS, pH 7.4) can take months to years depending on the required drug release duration, which is laborious and expensive. Therefore, a fast and reliable accelerated in vitro release method that can predict the real-time release in a short time is needed to save time and money [11,12]. Unfortunately, although several accelerated in vitro release methods have been developed for PLGA or PLA-based microspheres and preformed implants [12], to the best of our knowledge, there is no accelerated in vitro release method for injectable ISD systems reported in the literature yet. Our group has been developing ISD systems containing poly(lactide-*co*-glycolide) (PLGA) and polylactide (PLA) as an injectable dosage form for sustained release of levonorgestrel (LNG). These ISD systems could suppress the normal ovarian cycles in cottontop tamarins, a small New World non-human primate, for about six months after a single subcutaneous injection [6,13]. The primary purpose of this work was to develop an accelerated in vitro drug release method for LNG-loaded ISD systems for rapid screening/differentiation/optimization of long-acting contraceptives, and also provide some guidance that can be useful for the development of accelerated release methods for ISD systems in general.

The selection of appropriate stress conditions for accelerating the drug release is dependent on the nature and composition of the formulation, stability of the drug and release medium, release mechanisms, and requirements related to the sampling interval and duration of the study [14]. In this work, different combinations of four conditions of release media including: (a) temperature [11,14,15,16,17,18,19,20,21,22,23,24,25,26,27]; (b) presence of organic solvents such as alcohol, acetone, acetonitrile as co-solvents [12,15,28]; (c) presence of surfactants [18,28,29,30,31], and (d) pH [12,15,16] were used to develop an accelerated in vitro drug release method for screening ISD systems for long-term release of LNG for contraception. The correlations between the release kinetics and mechanisms of LNG from ISD systems under real-time and accelerated conditions were established.

## 2. Materials and Methods

### 2.1. Chemicals

Levonorgestrel was provided by Family Heath International (FHI360, Durham, NC, USA). Ester terminated poly(d,l-lactic-*co*-glycolic acid) (50:50) with an inherent viscosity (*iv*) of 0.55–0.75 dL/g was purchased from Lactel Absorbable Polymers (Birmingham, AL, USA). Ester terminated poly(d,l-lactide) with *iv* 0.40–0.50 and 0.60–0.80 dL/g were purchased from Lakeshore Biomaterials (Birmingham, AL, USA) and Evonik (Birmingham, AL, USA), respectively. The following chemicals and solvents were obtained from Fisher Scientific (Pittsburgh, PA, USA): triethyl citrate (TEC, 99%), *N*-methyl-2-pyrrolidnone (NMP), benzyl benzoate (BB), sodium chloride (crystalline/certified ACS), sodium hydrogen phosphate (98+%), potassium phosphate monobasic (extra pure 99+%), potassium chloride, water (HPLC grade), acetonitrile (HPLC grade), methanol (HPLC grade), ethanol (HPLC grade), and Tween 20.

### 2.2. Preparation of Injectable ISD Formulations

The formulations with composition listed in Table 1 were prepared as follows: LNG was weighed and added to the mixture solvent of NMP/TEC or NMP/BB (9:1 *v*/*v*) in a 20 mL vial. The vial was vortexed at 500 rpm until LNG dissolved. Weighted PLGA-50:50 and PLA polymers were then added and vortexed at 500 rpm until a clear uniform solution was obtained.

### 2.3. Real-Time (Long-Term) in Vitro Drug Release Study

The 96 and 64 formulations in Table 1 was injected through a syringe into a PTFE mold having a cylindrical cavity of 12.5 mm in diameter and 5 mm in depth at 400 and 160 µL, respectively, corresponding to a dose of 10 mg LNG. The mold was then transferred into a 500 mL jar, and 400 mL PBS (pH 7.4) release medium was carefully added into the jar. The jar was then placed into a 37 °C shaking incubator (MaxQ800, Thermo Scientific, Waltham, MA, USA) and constantly shaken at 50 rpm. At selected time points over a 4–5 month period, 1 mL of the release medium was collected for drug analysis using HPLC (see Section 2.5 for details), and then the entire medium was replaced with fresh PBS (pH 7.4) to maintain the sink condition. Each formulation was run in triplicate for the studies.

### 2.4. Accelerated (Short-Term) in Vitro Release Study

The formulations in Table 1 were prepared for the short-term in vitro release study the same way as for the long-term in vitro release study as described previously in Section 2.3, except for that the release conditions were the four conditions listed in Table 2 instead of PBS (pH 7.4) at 37 °C and the study was performed over a two week period instead of a four month period.

### 2.5. Drug Analysis

The collected release medium samples were analyzed for LNG content using HPLC (Shimadzu Scientific Instruments, Inc., Columbia, MD, USA) equipped with a BDS Hypersil C-18, 3 µm, 100 × 4.6 mm reverse phase column. Acetonitrile/0.01% formic acid in water (65:35 *v*/*v*) was used as a mobile phase. The flow rate was set at 0.8 mL·min^−1^ and the detection wavelength was 245 nm. The injection volume was 50 µL. The column temperature was kept at 25 °C. The standards for calibration were 0.070, 0.100, 0.250, 0.500, 0.750, 1.00, 2.50 µg·mL^−1^ of LNG dissolved in PBS (pH 7.4 correlation coefficient was *r*^2^ > 0.99).

## 3. Results and Discussion

### 3.1. Real-Time (Long-Term) in Vitro Release

**Figure 1:**
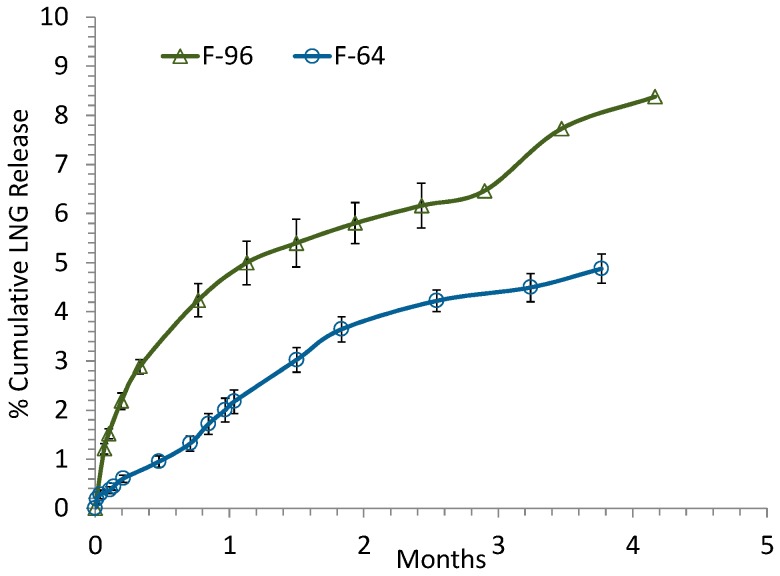
Real-time (long term) in vitro release of LNG from formulations 64 (open circle) and 96 (open triangle) in PBS medium (pH 7.4) at 37 °C.

The majority of in vitro drug release studies for the ISD systems have been conducted using variants of the sample-and-separate methodology. The studies were conducted by either injecting the formulation directly into a release medium or dialysis membrane which is then placed in a release medium, or holding the pre-formed gel or depot in a retainer including dialysis membrane [12]. However, the method of injecting the ISD formulation directly into the release medium usually generate depots of irregular shapes, which in turn results in unpredictable in vitro drug release. The dialysis method is dependent on the permeation of a drug across the dialysis membrane [32] and sometimes does not work for release of certain drugs. Therefore, to avoid the potential issues mentioned above, in this study we developed a simple and inexpensive in vitro release technique for the screening of the formulations using a cylindrical mold made of polytetrafluoroethylene (PTFE) with defined geometry (2.5 mm in diameter and 5 mm in depth) to study in vitro drug release kinetics from injectable ISD systems. The mold was immersed in the release medium in a glass jar, and the drug would diffuse out from the top surface into the release medium in a controlled manner. Though this method might not mimic well the in vivo drug release profile, it was sufficient and effective for achieving the objective of this study which is to identify an accelerated release condition that could help in the rapid screening of the formulations at early development stage. Figure 1 shows the cumulative release of LNG from depot formulations 96 and 64 in real time long term release condition (PBS medium, pH 7.4, 37 °C). Less than 10% of LNG release from the two depot formulations was observed over a 4–5 month period of time. The 96 formulation released LNG faster than the 64 formulation, which could be attributed to the differences in the composition of the formulations in terms of inherent viscosity of the PLA polymer (0.47 vs. 0.63), drug loading (2.5% vs. 6%) and solvent (BB vs. TEC). In the literature, it was explained that a polymer with lower inherent viscosity could get hydrated and swell better, and thus could release drug faster [33]. Due to the different drug loading (2.5% and 6%), 400 and 160 mL of the 96 and 64 formulations, respectively, were used to provide the same LNG dose at 10 mg for both formulations during the release study. The different volumes would be considered to possibly cause the different release kinetics too. However, our experimental data showed that the different volumes actually did not cause any difference in terms of release kinetics (data not shown).

### 3.2. Accelerated (Short-Term) in Vitro Release

**Figure 2:**
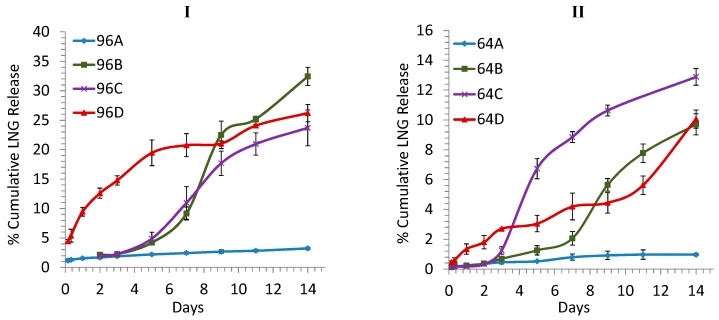
Short-term (accelerated) in vitro LNG release from formulations 96 (**I**) and 64 (**II**) at four accelerated conditions. (A) PBS, pH 7.4, 50 °C (filled diamond); (B) 25% ethanol in PBS, pH 7.4, 50 °C (filled square); (C) 25% ethanol in PBS, 2% Tween 20, pH 7.4, 50 °C (cross mark); (D) 25% ethanol in PBS, 2% Tween 20, pH 9, 50 °C (filled triangle). (*n* = 3).

As the real-time in vitro LNG release took up 4–5 months to be conducted, we explored four different release conditions as listed in Table 2 to develop an accelerated short-term (2 weeks) release method that would correlate well with the real time long term release so that it can be used for screening ISD systems for sustained release of LNG. The accelerated release profiles of the formulations 96 and 64 at four different conditions tested are shown in Figure 2(I,II), respectively.

Under elevated temperature alone (condition A: PBS, pH 7.4, 50 °C), Figure 2(I,II) show that only 3.2% and 0.97% of LNG was released from the formulations 96 and 64, respectively, during 14 days. These amounts were slightly higher than those corresponding amounts, ~2.97% (96) and ~0.47% (64), at 37 °C (Figure 1). When the release was conducted at elevated temperature, the drug release rate would increase as the drug diffusion and polymer degradation rate increased with temperature [11,15,16,17,18,19,20,21,22,34]. However, at elevated temperature, the polymer’s mobility also increased, which could cause a decrease of the drug release rate as the polymer movement could change the depot surface morphology and cause surface pore closure [11]. Possibly due to the competing effects mentioned above, the overall drug release rates from the two formulations did not increase too much when the temperature was increased from 37 to 50 °C. Further investigation is needed to test this hypothesis.

To achieve a higher LNG release rate, the temperature could be further increased. However, to avoid any potential high temperature related stability concern of the drug, we set the release temperature at 50 °C and explored the effects of additional parameters such as addition of organic solvent ethanol (condition B), and addition of ethanol and surfactant Tween 20 (condition C) in the release media on the drug release rate. Organic solvents such as alcohol, acetone, and acetonitrile added to the release medium as co-solvents were reported to accelerate drug release due to the swelling property of the hydro-alcoholic solvent which could cause the morphological changes such as surface pitting and pore formation of depots with time (hydro-alcoholic effect) [12,15,28]. As ethanol is more environmentally friendly than acetone and acetonitrile, we chose ethanol as a co-solvent for the accelerated release study. Surfactants such as Tween 20, Brij 35P, Triton X-100 could form micelles in the release medium to extract out the hydrophobic drug loaded in the depot so that the drug would be released faster from the depot [28,35,36]. In this accelerated release study, we chose ethanol as a co-solvent and Tween 20 as a surfactant. Figure 2(I, 96B),(II, 64B) showed that replacement of 25% of the release medium with ethanol did not cause much change in the LNG release rates from the two formulations 96 and 64 during the initial three days; the LNG release slowly increased during days 3 to 7, and the LNG release increased significantly during days 7 to 14 and about 9-fold on day 14. These results suggested that the hydro-alcoholic effect did not work so well to accelerate LNG release during the first 3 days. To study if further addition of surfactant would accelerate more LNG release, we added Tween 20 at 2 wt % in the release media and found that the addition of Tween 20 did not have much effect on the LNG release from formulations 96 and 64 during the first 7 and 5 days, respectively; decreased the LNG release from formulation 96 during 7–14 days, but it however increased the LNG release from formulation 64 dramatically after day 5 (Figure 2I(96C),II(64C)). The different effects of Tween 20 on LNG release from 96 and 64 formulations could be due to the differences in the porosities of the depots at condition C, which led to variation in the amount of Tween 20 micelles formed in the polymer matrix. The depot formed from 96 formulation probably generated more and faster pores than that from 64 formulation as the corresponding inherent viscosities of the PLA used for the 96 and 64 depot preparation were 0.47 and 0.63 dL/g, respectively. The more porous 96 depot may have allowed more surfactant to come inside the depot to form more micelles to hold more drug inside the depot. As a result, the addition of surfactant Tween 20 would have caused a significant amount of micelles formed inside the depot formed from 96 formulation so that LNG was released less from the depot under condition C than condition B after 7 days. The above interpretations of the results need further investigation.

As the three accelerated conditions A, B and C were unable to cause fast release during the first three days, we increased the pH of the release medium from 7 to 9 (condition D). Figure 2(I,II) show that the release of LNG from both the 96 and 64 formulations was significantly increased during the first three days under condition D in comparison to the other three conditions A, B, and C. The reason was probably due to the faster degradation of PLGA/PLA in the depot caused by alkaline pH 9, which was already well documented in the literature [37,38]. After 3 days, LNG was continuously released under condition D with amount about 8–10 times on day 14 than under condition A (pH 7.4); and higher and lower under condition D than condition C (pH 7.4, 25% ethanol, 2% Tween 20) after three days for formulations 96 and 64, respectively. These results were probably due to the balance of the polymer degradation and micelle formation inside and outside the depots.

The long-term goal of this study was to develop injectable formulations for sustained release of contraceptives for six months to years. Due to the intended long term release, the real time 4–5 month in vitro release in Figure 1 and the accelerated 2-week release in Figure 2 did not reach 80% LNG release, but instead reached <10% and <35%, respectively. According to FDA recommendation, an in vitro release should be conducted until the time point when 80% or higher or a plateau of drug release is attained [39]. However, the objective of the study was to identify an accelerated release condition that could be used to quickly differentiate formulations during early formulation screening stage rather than to develop a standard in vitro release protocol. The identified accelerated release condition D for a period of 2-week release study was sufficient and effective to achieve this objective.

### 3.3. Release Kinetics and Correlation between Accelerated and Real-Time Releases

To understand the release mechanisms of LNG from the depots made of formulations 96 and 64, all the release data in Figure 1 were fitted using empirical power law (Korsmeyer–Peppas Model, Equation (1)) [40] as shown below:

(1) $$\frac{M_{t}}{M_{\infty }}=kt^{n}$$

where *M_t_* and *M_∞_* are the amounts of drug released at time *t* and infinite (in this study the initial drug amount was used as *M_∞_*), respectively, *M_t_/M_∞_* is the fraction of drug released at time *t*, *k* is the rate constant related to diffusion coefficient, and *n* is the release exponent related to release mechanism. The correlations between n value and release mechanisms are: *n* < 0.5, non-Fickian diffusion due to increasing hydrophobicity with time; *n* = 0.5, Fickian diffusion; 0.5 < *n* < 0.1, anomalous (combination of Fickian and Case II diffusion) diffusion; *n* = 1, Case II diffusion due to polymer chain relaxation; and *n* > 1, Super Case II diffusion due to polymer chain relaxation [40,41]. The Equation (1) has been used frequently to analyze the drug release from several modified polymeric drug delivery systems including both swellable and non-swellable systems [41,42]. Table 3 lists the parameters k, n and coefficient of determination (*r*^2^) after fitting the release curves in Figure 1 and Figure 2 using Equation (1).

**Figure 3:**
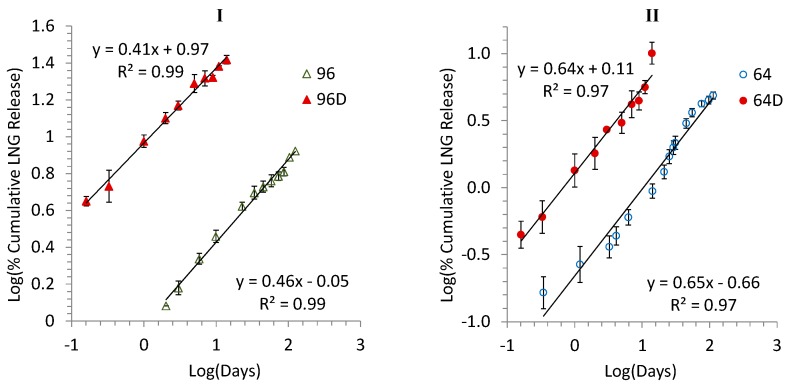
Power law kinetics (plot of log percent release vs. log of time in days) of formulations 96 (**I**; triangle) and 64 (**II**; circle) in real-time (open symbols) and accelerated (filled symbols) release condition D.

All the release curves in Figure 1 and Figure 2 could be fitted well by Equation (1) with *r^2^* ≥ 0.94 except for 64B and 64C which had slightly lower *r*^2^ values of 0.86 and 0.88, respectively. The *n* values ranged from 0.22 to 1.59 suggesting different release mechanisms under the four accelerated conditions for the formulations 96 and 64. The conditions A, B, and C either slightly increased (96A, 64A, 64B and 64C) or decreased (96B and 96C) the release rate constant k. The condition D significantly increased the k values by about ~10.2 and ~5.8 times for formulations 96 and 64, respectively, but well mimicked the release kinetics under real time condition for the two formulations with the similar fitting linearity (*r*^2^ = 0.99 for both 96D and 96; *r*^2^ = 0.97 for both 64D and 64) and release mechanisms (*n* = 0.41 and 0.46 for 96D and 96, respectively; *n* = 0.64 and 0.65 for 64D and 64, respectively). These results are visually illustrated in Figure 3I,II.

**Figure 4:**
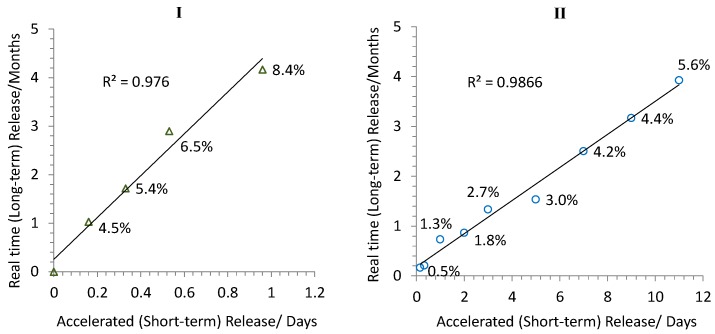
Correlation of accelerated (short-term) release at condition D with real-time (long-term) release testing of formulations 96 (**I**; triangle) and 64(**II**; circle).

To further correlate the short- and long-term LNG releases, the times required to achieve the same amounts of LNG to be released from the formulations 96 and 64 under condition D (days) and real-time (months) are shown in Figure 4 [26,34]. Excellent correlations between the release times under condition D and real-time were obtained with *r*^2^ = 0.98 and 0.99 for formulation 96 and 64, respectively. These results suggested that the condition D was able to reduce the release time significantly compared to the real-time, and allows for the prediction of LNG long-term release from ISD systems in a short time.

## 4. Conclusions

In summary, elevated temperature, addition of alcohol and surfactant, and change of the pH of the releasing medium to be basic could result in an increase of the drug release rate of in situ forming depot systems, due to higher degradation of the depot polymers, increase of the porosity of the depot matrices, and thus fast diffusion of the drug out of the depots. The developed accelerated release condition D (25% ethanol in PBS, 2% Tween 20, pH 9, 50 °C) shortened the 4–5 month real-term LNG release from formulations 96 and 64 in PBS (pH 7.4) at 37 °C to days without changing the drug release mechanisms. The developed accelerated release method has significance not only in helping rapid screening of formulations for the development of ISD-based contraceptives containing LNG, but also for providing guidance for the development of accelerated in vitro release testing methods for ISD systems in general.

## Figures and Tables

**Table 1:** The composition of formulations for in vitro release study.

Formulation #	PLGA 50:50 wt %	PLA wt %	LNG wt %	Solvent (9:1) wt %
96	4.1 (*iv* − 0.63)	20.3 (*iv* − 0.47)	2.5	73.1 NMP/BB (9:1)
64	4.7 (*iv* − 0.63)	18.8 (*iv* − 0.63)	6	70.5 NMP/TEC (9:1)

**Table 2:** Release testing conditions used for accelerated in vitro release method development.

Condition	PBS (% *v*/*v*)	Ethanol (% *v*/*v*)	Tween 20 (% *w*/*v*)	pH	Temperature (°C)
A	100%	0	0	7.4	50
B	Adjusted to 100%	25%	0	7.4	50
C	Adjusted to 100%	25%	0.5%	7.4	50
D	Adjusted to 100%	25%	0.5%	9.0	50

**Table 3:** Fitting parameters determined by the linear regression of log(*M_t_*/*M*_∞_) against log*t* in Equation (1) for the LNG release from the depots made of formulations 96 and 64 under real time and four accelerated release conditions.

Formulation (Release Condition)	Power Law (Korsmeyer–Peppas Model)		
	*r^2^* (Coefficient of Determination)	*k* (Rate Constant)	*n* (Release Exponent)
96(A)	0.96	1.62	0.22
96(B)	0.94	0.50	1.59
96(C)	0.97	0.57	1.15
96(D)	0.99	9.23	0.41
96 (long-term)	0.99	0.90	0.46
64(A)	0.99	0.26	0.52
64(B)	0.86	0.41	0.97
64(C)	0.88	0.49	1.24
64(D)	0.97	1.28	0.64
64 (long-term)	0.97	0.22	0.65
